# Learning Transcriptional Regulatory Relationships Using Sparse Graphical Models

**DOI:** 10.1371/journal.pone.0035762

**Published:** 2012-05-07

**Authors:** Xiang Zhang, Wei Cheng, Jennifer Listgarten, Carl Kadie, Shunping Huang, Wei Wang, David Heckerman

**Affiliations:** 1 Microsoft Research, Los Angeles, California, United States of America; 2 Case Western Reserve University, Cleveland, Ohio, United States of America; 3 University of North Carolina at Chapel Hill, Chapel Hill, North Carolina, United States of America; University of Sheffield, United Kingdom

## Abstract

Understanding the organization and function of transcriptional regulatory networks by analyzing high-throughput gene expression profiles is a key problem in computational biology. The challenges in this work are 1) the lack of complete knowledge of the regulatory relationship between the regulators and the associated genes, 2) the potential for spurious associations due to confounding factors, and 3) the number of parameters to learn is usually larger than the number of available microarray experiments. We present a sparse (L1 regularized) graphical model to address these challenges. Our model incorporates known transcription factors and introduces hidden variables to represent possible unknown transcription and confounding factors. The expression level of a gene is modeled as a linear combination of the expression levels of known transcription factors and hidden factors. Using gene expression data covering 39,296 oligonucleotide probes from 1109 human liver samples, we demonstrate that our model better predicts out-of-sample data than a model with no hidden variables. We also show that some of the gene sets associated with hidden variables are strongly correlated with Gene Ontology categories. The software including source code is available at http://grnl1.codeplex.com.

## Introduction

Transcriptional regulatory networks govern the expression levels of thousands of genes as part of a diverse biological processes. Regulatory proteins called transcription factors (TF) are the main players in the regulatory network. TFs bind to promoter regions at the start of other genes and thereby initiate or inhibit gene expression. Determining accurate models for transcriptional regulatory interactions is an important challenge in computational biology. With the development of high-throughput DNA microarray technologies, it is possible to simultaneously monitor the expression levels of essentially all genes. Extensive research has been done to build quantitative regulatory models by associating gene expression levels (see [1]–[3] for reviews).

One challenge in this work is that not all TFs have been identified and the regulatory relationship between TFs and their associated genes may not be available (except for some well studied model organisms such as yeast). Another challenge is the potential for spurious associations between regulators and affected genes due to confounding factors such as expression heterogeneity [4]–[8]. Moreover, in large scale genome-wide expression datasets, the number of genes (or probs) is usually much larger than the number of samples. This is the so called “large 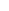 small 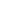
” problem [9], [10]. Feature selection is required when analyzing such datasets.

Various methods have been proposed to learn the regulatory relationship between TFs and their associated genes [11]–[16]. Assuming that the (partial) knowledge of the network topology between TFs and genes is available, network component analysis [11], [15] aims to reconstruct signals from the regulators and their strengths of influence on each genes. However, such knowledge may not be always available, e.g., for human. Similarly, in [13], [14], methods have been proposed to infer the TF activities (concentration levels) assuming the TF-gene relationship is known. The work in [12] does not assume a known regulatory network, and tries to reconstruct one from sequence and array data. The proposed methods was applied to yeast datasets. The goal is different from ours, which is to reconstruct the regulatory network from the microarray data without the sequence information. Clustering approaches have also been developed to analyze gene expression data [17]–[20]. These methods partition samples into groups according to the expression patterns of genes in different groups. The TF information is not used in these algorithms.

**Figure 1:**
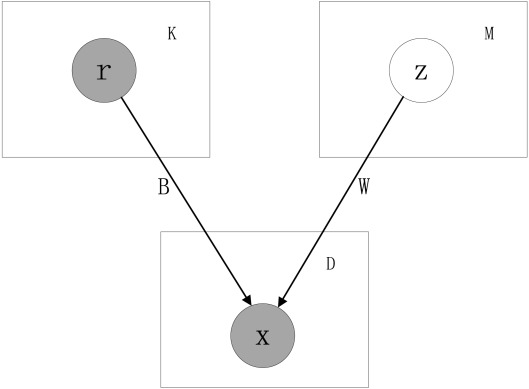
The graphical model Known and potential TFs are assumed to be mutually independent. Regulated genes are assumed to be mutually independent given the TFs.

In this paper, we propose a linear-Gaussian graphical model to address the challenges in learning regulatory relationships. Our model consists of two layers of nodes as shown in Figure 1. The upper layer nodes include the set of known/putative TFs and a set of hidden variables. The hidden variables are used to model possible unknown TFs and confounding factors. The lower layer represents the remaining genes that are not included in the upper layer. The nodes are connected via arcs from the upper layer nodes to the lower layer nodes. The expression levels of a node (gene) in the lower layer are modeled as a linear function of the expression levels of the upper layer nodes–that is, known TFs and hidden factors. Note that graphical model has also recently been applied to find expression quantitative trait loci [21].

To learn the parameters of the model from data, which is usually of high dimension and low sample size, we use L1 regularization as is done in [22] (see also [23]–[25]). This approach yields a sparse network, where a large number of association weights are zero [26]. In gene regulatory networks, the number of TFs is much smaller than the number of transcribed genes, and most genes are regulated by a small number of TFs. The matrix that describes the connections between the transcription factors and the regulated genes is expected to be sparse. Thus L1 regularization is a natural choice for this setting.

We apply our model to large scale human gene expression data and show that our model has better prediction accuracy than do other alternatives. We examine each gene set defined by those in the lower layer connected to a single hidden variable in the upper layer. We find that some of these gene sets are strongly correlated with GO categories, suggesting that the hidden variables at least in part represent unknown TFs. The software including source code is publically available at http://grnl1.codeplex.com.

## Methods

### Linear Regression and Probabilistic PCA

Our model can be thought of as a combination of linear regression and probabilistic principal component analysis (PPCA) [27] with L1 regularization. In this subsection, we briefly review these two approaches.

Throughout the paper, we assume that all vectors are column vectors. Let 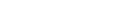 represent the 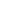 known/putative TFs (e.g., [28]), and 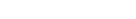 represent the 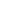 genes in the dataset. Note that the two sets 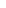 and 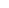 are disjoint–that is, 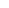 include the genes that are not TFs. This restriction is added so that the resulting graph is acyclic and therefore amenable to straightforward estimation techniques. The linear regression model assumes that the expression level of a gene 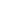 can be represented by a linear function of the expression levels of the TFs.
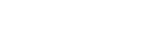
where 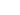 (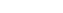) is the coefficient that quantifies the strength of the TF 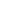 to initiate (positive) or suppress (negative) the regulation of gene 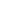, 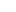 is a translation factor, and  is the additive noise of Gaussian distribution with zero-mean and standard deviation 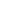
–that is, 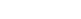


The idea of PPCA is similar to that of linear regression. The difference is that the expression level of a gene 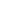 is modeled as a linear function of the expression levels of a set of hidden (unobserved) variables 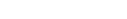:
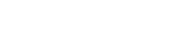
where 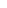 has a zero-mean, unit-variance Gaussian distribution.

### Our Model

To incorporate both known/putative TFs and unknown factors, our model combines linear regression and PPCA. We model the expression level of a gene to be a linear function of the expression levels of both known/putative TFs and hidden factors.
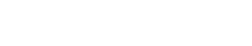
A graphical representation of the model is shown in Figure 1. It has two layers. The upper layer consists of random (vector) variable 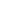 representing TFs, and 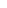 representing hidden factors. The factors are assumed to mutually independent (although, because the known/putative factors are observed, any dependencies among them do not affect the predictive ability of the model). The lower layer contains the random variable 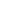 representing the genes regulated by the upper layer nodes. These regulated genes are assumed to be mutually independent given the regulators in the upper layer.

Next, we use multivariate notation to formalize and derive the likelihood function of our model. Let 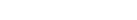, 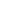 be the 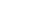 matrix with the 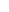-th row being 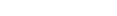, and 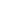 be the 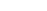 matrix with the 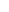-th row being 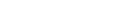. We have that
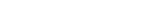
where 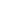 is the identity matrix. Let the prior distribution over latent variable **z** be given by a zero-mean unit-covariance Gaussian



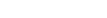
The conditional distribution of the observed variable **x**, conditioned on the value of the latent variable 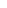, is also Gaussian, of the form



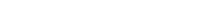
Integrating out latent variable **z**,



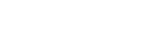
the marginal distribution is again Gaussian



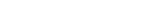
where the 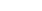 covariance matrix **C** is defined by



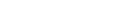
The complexity of inverting **C** is 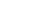 instead of 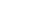




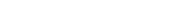
where the 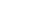 matrix **M** is defined by



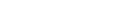
Let **R** = {**r** 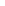} and **X** = {**x** 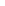} be the sets of 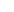 observed data points. The loss function (negative log likelihood function) is



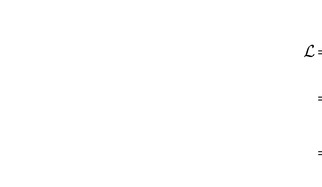



The parameter space in our model is 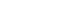. Since only a small fraction of the candidate TFs are expected to be true regulators for any given gene, most of the weights in 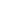 and 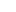 should be set to zero to indicate non-regulation. L1 regularization is a well known approach for effective feature selection. In this approach, we add a penalty to the objective function that automatically pushes the elements in the parameter space to be zero. It has shown experimentally and theoretically to be capable of learning good models when most features are irrelevant [26]. The new objective function with L1 regularization is of the form
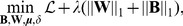
(1)where 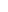 is a tuning parameter that can be determined using cross validation, which will be discussed later.

### Optimization

To optimize the likelihood function with L1 norm, we use the Orthant-Wise Limited-memory Quasi-Newton (OWL-QN) algorithm described in [29]. The OWL-QN algorithm minimizes functions of the form
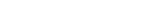
where *loss* is an arbitrary differentiable loss function, and 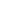 is the L1 norm of the weight (parameter) vector. It is based on the L-BFGS Quasi-Newton algorithm [30], with modifications to deal with the fact that the L1 norm is not differentiable. The algorithm is proven to converge to a local optimum of the parameter vector. The algorithm is very fast, and capable of scaling efficiently to problems with millions of parameters. Thus it is a good option for our problem where the parameter space is large when dealing with large scale genome-wide gene expression data.

Besides the loss function, and the penalized parameters, the OWL-QN algorithm also needs the gradient of the loss function, which (without detailed derivation) is
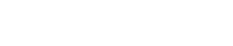


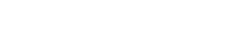
where



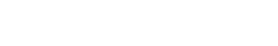


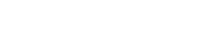


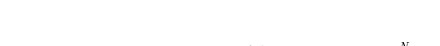
The number of hidden variables 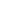 and the L1 penalty 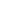 are determined by two-fold cross validation within a wrapper used to evaluate out-of-sample prediction (see Evaluation). Only two folds are used at this stage to lessen the computational burden.

We note that sparse PCA is not convex [31]. Nonetheless, when we applied the optimization program with 10 random parameter initializations for give different models, the program converged to the same solution for each condition.

## Results and Discussion

### Data Set

The gene expression data is taken from 1109 human liver samples. Each RNA sample was profiled on a custom Agilent 44,000 feature microarray composed of 39,296 oligonucleotide probes targeting transcripts representing 34,266 known and predicted genes, including high-confidence, non-coding RNA sequences. The gene expression data was originally collected to characterize the genetic architecture of gene expression in human liver [32]. The expression data was processed using the median imputation method as in [32]. All microarray data associated with the human liver cohort were previously deposited into the Gene Expression Ominbus (GEO) database [33] under accession number GSE24335. The set of known and putative TFs is taken from [28], which is publicly available from http://hg.wustl.edu/lovett/TF_june04table.html. The total number of such TFs is 1660.

### Evaluation

We evaluated three models: (1) one with hidden variables, (2) one with no hidden variables, and (3) a reference model that assumes the non-TF genes are mutually independent (i.e., a model with no top layer in the corresponding graph). We evaluated the models by measuring out-of-sample log likelihoods via ten-fold cross validation. More specifically, we partition the samples into 10 subsets of equal size. In each fold, we use samples in 9 subsets as training data and test the learned model in the remaining 1 subset of samples. By measuring out-of-sample versus in-sample predictions, we avoid rewarding models that over fit the data. Within each cross, optimal values for 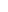 were determined with two-fold cross validation. In one fold, the optimal value for 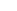 (the number of hidden variables) was determined to be 20; and we used this value for the remaining nine folds.

**Figure 2:**
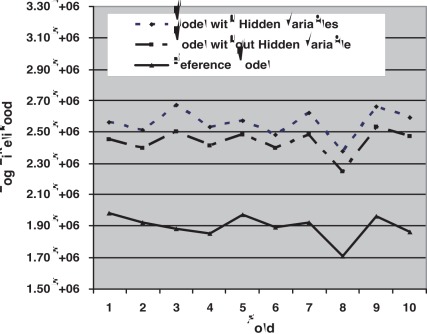
Out-of-sample prediction accuracy of the three models across the 10 folds of the data.

Figure 2 shows the model log-likelihoods of the out-of-sample predictions across the 10 folds of the data. As can be seen from the figure, the model with hidden variables always outperforms the model without hidden variables. Assuming the log likelihoods are independent (which is roughly the case as there are only 10 folds in the cross validation), the difference in predictive ability is significant (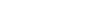 via a paired Wilcoxon signed rank test). Similarly, the model without hidden variables predicts significantly better than does the reference model (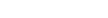).

### GO Enrichment Analysis for Gene Sets Associated with Hidden Variables

Hidden variables can model the effect of unknown regulators or hidden confounders. To better understand the effect of the hidden variables, we look for correlations between genes associated with a given hidden variable and sets of genes in GO categories (Biological Process Ontology) [34].The GO categories are downloaded from website for gene set enrichment analysis (GSEA) http://www.broadinstitute.org/gsea/. In particular, for each gene set 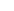, we identify the GO category whose set of genes is most correlated with 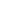. We measure correlation via a 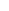-value determined by application of Fisher’s exact test. Since multiple gene sets 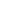 need to be examined, the raw p-values need to be calibrated because of the multiple testing problem [35]. To compute calibrated 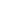-values for each 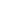, we perform a randomization test, wherein we apply the same test to 1000 randomly created gene sets that have the same number of genes as 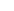.

In Table 1, each row represents the gene set associated with a hidden variable. The calibrated p-values for the gene sets associated with hidden variables are listed in the second column in the table. The third column shows the false discovery rate (FDR) [36] of the gene sets. As can be seen from the Table, with an FDR significance threshold 0.05, nine of the twenty gene sets are significant. These nine hidden variables may represent the joint effect of unknown TFs. The remaining hidden variables may correspond to hidden confounders.

**Table 1:** GO enrichment analysis of the gene sets associated with hidden variables.

Gene Set Size	Raw p-value	Adjusted p-value	FDR	GO Categories
19649	1.17×10^−15^	0	0	cellular protein metabolic process
19431	2.31×10^−13^	0	0	protein metabolic process
22301	1.71×10^−10^	0	0	transport
23608	2.53×10^−9^	0	0	transport
20500	9.47×10^−9^	0	0	cellular protein metabolic process
26332	1.55×10^−8^	0	0	transport
21264	2.20×10^−5^	0.001	0.003	response to chemical stimulus
19395	1.87×10^−5^	0.004	0.01	organic acid metabolic process
21098	1.51×10^−4^	0.01	0.022	organic acid metabolic process
29240	2.03×10^−3^	0.026	0.052	synaptic transmission
20199	3.76×10^−4^	0.03	0.054	positive regulation of phosphate metabolic process
24175	1.04×10^−3^	0.048	0.08	phosphoinositide mediated signaling
17480	6.73×10^−4^	0.064	0.1	cation homeostasis
20331	9.45×10^−4^	0.07	0.1	digestion
22477	1.29×10^−3^	0.075	0.1	locomotory behavior
22644	2.74×10^−3^	0.204	0.255	organic acid transport
18732	4.00×10^−3^	0.393	0.462	positive regulation of t_cell proliferation
16294	7.86×10^−3^	0.707	0.786	inorganic anion transport

The first column of Table 1 shows the sizes of the gene sets associated with hidden variables. As can be seen, each gene set covers a large number of genes, despite the use of an L1 penalty that tends to drive many association weights to zero. This result indicates that the hidden variables may model the effects that influence many different genes.

**Figure 3:**
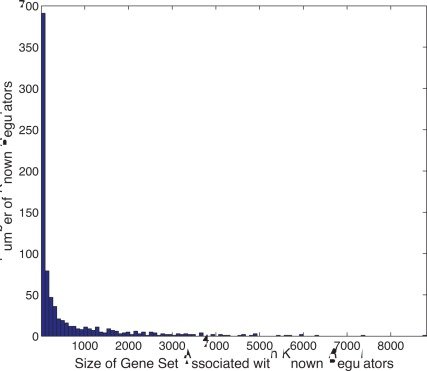
Histogram of sizes of the gene sets associated with known and putative regulators.

Among the gene sets associated with known/putative regulators, there are 803 gene sets with size greater or equal to 5. The maximum size is 8820. Figure 3 shows the histogram of sizes of these gene sets. It can be seen from the figure that the gene sets associated with known/putative regulators are much smaller than those associated with hidden variables. This is reasonable since real regulators are expected to regulate a relatively small subset of genes. On the other hand, the large sizes of the gene sets associated with hidden variables indicate that the hidden variables are useful in modeling confounding factors that may effect most of the genes.

### Comparison to Network Component Analysis Method

Our method aims to learn the transcriptional regulatory relationship without any prior knowledge of the network topology. As discussed in the Introduction section, various methods have been proposed to learn transcription factor activity assuming that the regulatory network topology is known [11]–[16]. Among the existing methods, Network Component Analysis (NCA) is a widely used approach. NCA aims at decomposing gene expression matrix 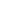 into two matrices 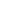 and 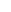, such that 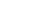, where 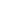 represents the connectivity network, and 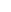 presents the transcriptional factor activities (TFA). The connectivity matrix 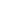 is a required input of NCA. A nonzero value indicates there is an edge from a TF to a gene, and zero value indicates there is no edge between them. The nonzero values in the input matrix 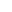 can be random. The algorithm automatically learns the optimized 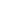 and 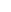. The zero entries in 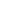 remain unchanged. That is, the structure of the regulatory network does not change. Therefore, NCA is mainly used to infer the TF activities with known network structure.

The NCA algorithm needs three criteria to ensure the decomposition to be unique [11]. First, the connectivity matrix 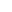 must have full-column rank. Second, when a node in the regulatory layer is removed along with all of the output nodes connected to it, the resulting network must be characterized by a connectivity matrix that still has full-column rank. This implies that each column of A must have at least 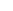-1 zeros, where 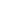 is the number of TFs. Third, matrix 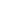 must have full row rank.

To apply NCA to infer the regulatory structure, we use a random matrix as the input matrix 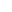, of which 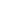-1 random elements in each column are set to 0. This is needed in order to satisfy the three criteria required by NCA. For the fairness of comparison, after the connectivity structure is learned by NCA, we remove the edges with small weights so that the number of remaining edges is equal to that of our model.

We apply GO enrichment analysis on the gene sets learned by NCA and our method. Table 2 shows the average raw p-value of the gene sets and the number of significant gene sets (with significance level 0.05 after correction for multiple testing). As can been seen from the table, the average raw p-value of our model is much less than that of NCA. Moreover, our model identified more significance gene sets than did NCA. The main reason for this difference is that NCA requires prior knowledge about the regulatory structure. Our model dose not have this assumption and tries to reconstruct the regulatory structure from the expression values of the TFs and genes.

**Table 2:** GO enrichment analysis of the gene sets identified by NCA and our model.

Method	Average raw p-value	Number of gene sets with calibrated p-values 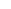 0.05
NCA	0.024	5
Our model	0.007	219

### Conclusion

Reconstructing gene transcriptional regulatory networks is a central problem in computational systems biology. Challenging issues include the incorporation of knowledge about TFs and modeling unknown TFs and confounders. We have developed a probabilistic graphical model that includes the known TFs as observed variables, uses hidden variables to model unknown TFs and confounders, and uses L1 regularization to address the high dimensionality and relatively low sample size of the data. Using human gene expression data, we have shown that the proposed model predicts significantly better than does the model without hidden variables. In addition, we have found that some of gene sets corresponding to hidden variables have significant correlations with GO categories, suggesting that the hidden variables at least in part represent unknown TFs.

## References

[1] Schlitt T, Brazma A (2007). Current approaches to gene regulatory network modelling.. BMC Bioinformatics 8(suppl.

[2] Hache H, Lehrach H, Herwig R (2009). Reverse engineering of gene regulatory networks: A comparative study..

[3] Lee WP, Tzou WS (2009). Computational methods for discovering gene networks from expression data.. Briefings in Bioinformatics.

[4] Leek JT, Storey JD (2007). Capturing heterogeneity in gene expression studies by surrogate variable analysis..

[5] Kang HM, Ye C, Eskin E (2008). Accurate discovery of expression quantitative trait loci under confounding from spurious and genuine regulatory hotspots.. Genetics.

[6] Michaelson JJ, Loguercio S, Beyer A (2009). Detection and interpretation of expression quantitative trait loci (eQTL).. Methods.

[7] Stegle O, Kannan A, Durbin R, Winn J (2008). Accounting for non-genetic factors improves the power of eqtl studies. In: Vingron M, Wong L, editors, RECOMB.. Springer, volume 4955 of Lecture Notes in Computer Science,.

[8] Gilad Y, Rifkin SA, Pritchard JK (2008). Revealing the architecture of gene regulation: the promise of eQTL studies.. Trends Genet.

[9] Hastie T, Tibshirani R, Friedman JH (2001). The elements of statistical learning: data mining, inference, and prediction..

[10] Bishop CM (2006). Pattern Recognition and Machine Learning..

[11] Liao J, Boscolo R, Yang YL, Tran LM, Sabatti C (2003). Network component analysis: Reconstruction of regulatory signals in biological systems.. PNAS.

[12] Sabatti C, James G (2006). Bayesian sparse hidden components analysis for transcription regulation networks.. Bioinformatics.

[13] Sanguinetti G, Lawrence N, Rattray M (2006). Probabilistic inference of transcription factor concentrations and gene-specific regulatory activities.. Bioinformatics.

[14] Boorsma A, Lu X, Zakrzewska A, Klis F, Bussemaker HJ (2008). Inferring condition-specific modulation of transcription factor activity in yeast through regulon-based analysis of genomewide expression.. PLoS One.

[15] Chang CQ, Ding Z, Hung YS, Fung P (2008). Fast network component analysis (fastnca) for gene regulatory network reconstruction from microarray data.. Bioinformatics.

[16] Wang K, Saito M, Bisikirska B, Alvarez M, Lim WK (2009). Genome-wide identification of posttranslational modulators of transcription factor activity in human b cells.. Nat Biotech.

[17] Eisen M, Spellman P, Brown P, Botstein D (1998). Cluster analysis and display of genome wide expression patterns.. Proceedings of the National Academy of Sciences.

[18] Ben-Dor A, Shamir R, Yakhini Z (1999). Clustering gene expression patterns.. Journal of Computational Biology.

[19] Alon U, Barkai N, Notterman D, Gish K, Ybarra S (1999). Broad patterns of gene expression revealed by clustering analysis of tumor and normal colon tissues probed by oligonucleotide arrays.. Proceedings of the National Academy of Sciences.

[20] Tavazoie S, Hughes J, Campbell M, Cho R, Church G (1999). Systematic determination of genetic network architecture.. Nature genetics.

[21] Parts L, Stegle O, Winn J, Durbin R (2011). Joint genetic analysis of gene expression data with inferred cellular phenotypes.. PLoS Genetics.

[22] Lee SI, Dudley A, Drubin D, Silver PA, Krogan NJ (2009). Learning a prior on regulatory potential from eqtl data.. PLoS Genet.

[23] Tibshirani R (1996). Regression shrinkage and selection via the lasso.. J Royal Statist Soc B.

[24] Efron B, Hastie T, Johnstone I, Tibshirani R (2004). Least angle regression.. Annals of Statistics.

[25] Guan Y, Dy JG (2009). sparse probabilistic principal component analysis..

[26] Ng A (2004). Feature selection, l1 vs..

[27] Tipping ME, Bishop CM (1999). Probabilistic principal component analysis.. Journal of the Royal Statistical Society.

[28] Messina D, Glassock J, Gish W, Lovett M (2004). An orfeome-based analysis of human transcription factor genes and the construction of a microarray to interrogate their expression.. Genome Research.

[29] Andrew G, Gao J (2007). Scalable training of l1-regularized log-linear models..

[30] Nocedal J, Wright SJ (2006). Numerical optimization..

[31] Zou H, Hastie T, Tibshirani R (2006). Sparse principal component analysis.. Journal of Computational and Graphical Statistics.

[32] Schadt EE, Molony C, Chudin E, Hao K, Yang X (2008). Mapping the genetic architecture of gene expression in human liver.. PLoS biology.

[33] Edgar R, Domrachev M, Lash AE (2002). Gene expression omnibus: Ncbi gene expression and hybridization array data repository.. Nucleic Acids Res.

[34] The Gene Ontology Consortium (2000). Gene ontology: tool for the unification of biology.. Nature Genetics.

[35] Westfall PH, Young SS (1993). Resampling-based Multiple Testing..

[36] Benjamini Y, Hochberg Y (1995). Controlling the false discovery rate: a practical and powerful approach to multiple testing.. Journal of the Royal Statistical Society Series B (Methodological),.

